# Remediation of Lead-Contaminated Water by Red Yeast and Different Types of Phosphate

**DOI:** 10.3389/fbioe.2022.775058

**Published:** 2022-03-21

**Authors:** Da Tian, Xiaohui Cheng, Liyan Wang, Jun Hu, Ningning Zhou, Jingjing Xia, Meiyue Xu, Liangliang Zhang, Hongjian Gao, Xinxin Ye, Chaochun Zhang

**Affiliations:** ^1^ Anhui Province Key Lab of Farmland Ecological Conservation and Pollution Prevention, College of Resources and Environment, Anhui Agricultural University, Hefei, China; ^2^ Research Centre of Phosphorus Efficient Utilization and Water Environment Protection Along the Yangtze River Economic Belt, Anhui Agricultural University, Hefei, China; ^3^ Anhui Engineering and Technology Research Center of Intelligent Manufacture and Efficient Utilization of Green Phosphorus Fertilizer, Anhui Agricultural University, Hefei, China; ^4^ Key Laboratory of JiangHuai Arable Land Resources Protection and Eco-restoration, Ministry of Natural Resources, Hefei, China

**Keywords:** EPS, Pb remediation, phosphate, phosphogypsum, red yeast

## Abstract

*Rhodotorula* *mucilaginosa* (Rho) can secrete large amounts of extracellular polymeric substances (EPS) to resist lead (Pb) toxicity. Phosphate is an effective material for the remediation of Pb. This study explored the Pb remediation by the combination of Rho and different types of phosphate in water. To do so, four phosphates, namely, ferric phosphate (FePO_4_, Fe-P), aluminum phosphate (AlPO_4_, Al-P), calcium phosphate [Ca_3_(PO_4_)_2_, Ca-P], and phosphogypsum (PG) were employed along with Rho. Compared with Rho application, the addition of phosphate significantly promoted the secretion of EPS by Rho (21–25 vs 16 mg). The formed EPS-Pb contributes to the Pb immobilization in the combination of Rho and phosphate. After 6 days of incubation, Rho + phosphate treatments immobilized over 98% of Pb cations, which is significantly higher than Rho treatment (94%). Of all Rho + phosphate treatments, Ca-P and PG-amended Rho had higher secretion of EPS, resulting in higher Pb removal. Nevertheless, PG had the highest efficiency for Pb removal compared with other phosphates, which reached 99.9% after 6 days of incubation. Likewise, new Pb minerals, such as pyromorphite and lead sulfate, only appeared in Rho + PG treatment. Altogether, this study concludes on the combined application of Rho and phosphate as an efficient approach to promote Pb remediation, particularly using PG waste.

## Introduction

Worldwide, lead (Pb) contamination has been recognized as a growing concern, especially in water bodies (Gadd, 1993; Zeng et al., 2017). The contamination of Pb in water mainly comes from waste and wastewater discharged by industrial and mining enterprises such as lead storage batteries, metal mining, and other anthropogenic activities (Hou et al., 2018; Shen et al., 2018; Tian et al., 2019). Unlike organic pollutants, Pb is a stable and hardly biodegradable contaminant in the environment (Radoicic & Raicevic, 2008; Rhee et al., 2012). High Pb toxicity in water would damage the aquatic animals’ development. More importantly, Pb accumulates continuously in organisms and threatens human health through food consumption (Cao et al., 2009; Hou et al., 2017; Shen et al., 2018). Hence, the Pb pollutants in water should be highly valued.

Phosphate is an effective material for the remediation of Pb in water (Li et al., 2016). Phosphorus (P) released from phosphate can react with Pb cation (Pb^2+^) to form the highly insoluble pyromorphite (Pyro) [Pb_5_(PO_4_)_3_X] (X = F, OH, or Cl), which has a very low solubility (Li et al., 2016). Geological apatite [Ca_10_(PO_4_)_6_(F, OH, Cl)_2_], calcium phosphate (Ca-P, Ca_3_(PO_4_)_2_), aluminum phosphate (Al-P, AlPO_4_), and ferric phosphate (Fe-P, FePO_4_) are the most common phosphate species in nature (Tian et al., 2021b). In addition, the by-products of phosphogypsum (PG) in P chemical industry also contain residual phosphate (Saadaoui et al., 2017). Despite their high affinity for binding Pb, the use of these phosphate species is limited due to their low solubility. To respond to this challenge, microorganisms can be applied together with phosphate to promote Pb remediation *via* the secretion of secondary metabolites such as low-molecular weight organic acid (LMWOA) and extracellular polymeric substances (EPS) (Coutinho et al., 2012; Frisvad et al., 2018).

LMWOAs and EPS not only enhance P release from phosphate to form insoluble Pb minerals, but they also react with Pb (Li et al., 2019; Tian et al., 2019). For example, phosphate-solubilizing fungi (PSF, e.g., *Aspergillus niger*) can secrete large amounts of oxalic acid to dissolve apatite and promote the transfer of Pb^2+^ to pyro and lead oxalate (Li et al., 2016). However, both the types of phosphate and fungi would affect the efficiency of Pb remediation (Shen et al., 2018; Tian et al., 2019). On the one hand, Pb remediation is influenced by the dissolving capacity of various phosphate species (Tian et al., 2021b). On the other hand, fungal activity is affected by different levels of Pb toxicity (Li et al., 2019; Tian et al., 2019; Jiang et al., 2020). Hence, it is of major importance to select suitable phosphates and fungi for water Pb remediation.

Red yeast, a widely available fungus in nature, has a high growth rate and strong environmental resistance, especially in wastewater environments (Kot et al., 2019a; Naveed et al., 2019). Unlike PSF, red yeast mainly secretes large amounts of EPS, rather than organic acids (Gientka et al., 2015). The EPS contains a variety of organic components that, in turn, have a highly branched chemical structure and functional groups, such as hydroxyl and carboxyl groups (Kot et al., 2019b; Rahbar Saadat et al., 2021). This spatial structure and complex composition can ensure that red yeast adsorbs and chelates with Cd^2+^ and Pb^2+^, which reduces the toxicity of heavy metals (Li et al., 2019). For example, the red yeast *Rhodotorula mucilaginosa* (Rho) can resist high Pb levels (2,500 mg/L) and adsorb Pb cations to form EPS-Pb (Jiang et al., 2020). Therefore, the combination of Rho and phosphate would be a feasible and effective way in Pb remediation.

In this study, we investigated the combined application of Rho and four types of phosphates for Pb remediation. The water-soluble P and Pb concentrations were analyzed by inductively coupled plasma optical emission spectrum (ICP-OES). The composition of precipitates was analyzed by X-ray diffraction (XRD). The morphology of Rho and its mineral composition were observed by using a scanning electron microscope–energy dispersive spectrometer (SEM-EDS).

## Materials and Methods

### Red Yeast Incubation

Red yeast *Rhodotorula mucilaginosa* (Rho) was isolated from orchard rhizosphere soil (CGMCC No.16597, Nanjing Agricultural University) (Li et al., 2019). Before the experiment, Rho was inoculated to a potato dextrose broth (PDB) medium (sterilized at 121°C for 20 min) and shaken for 48 h at 28°C, 180 rpm.

### Phosphate Preparation

Four phosphates, such as ferric phosphate (FePO_4_, Fe-P), aluminum phosphate (AlPO_4_, Al-P), calcium phosphate [Ca_3_(PO_4_)_2_, Ca-P], and phosphogypsum (PG), were used for the remediation of Pb by Rho. Fe-P, Al-P, and Ca-P were supplied by Shanghai Macklin Biochemical Co. Ltd. PG was collected from a phosphate fertilizer production plant in Anhui Province, China. Before the experiment, all phosphate species were dried at 65°C for 24 h.

### Pb Remediation by Rho and Phosphate

The Pb contamination in water was prepared by Pb(NO_3_)_2_ powder (Xilong Scientific Ltd.). The initial Pb concentration in the medium was adjusted to 1,000 mg/L. Five treatments were performed in this experiment, that is, Rho, Rho + Fe-P, Rho + Al-P, Rho + Ca-P, and Rho + PG. Before the incubation, the 0.08 g Pb(NO_3_)_2_ powder and 0.5 g phosphate were added to 150-ml Erlenmeyer flasks with 50 ml PDB medium (sterilized at 121°C for 20 min) in a sterile environment. Then, 0.5 ml Rho suspension was added to each treatment. After sealing with parafilm (BS-QM-003, Biosharp), the flasks were incubated at 180 rpm, 28°C in a sterile condition. After incubating for 2, 4, and 6 days, the PDB medium was collected into 50-ml centrifugal tubes and centrifuged for 6 min at 5,000 rpm. Then, the supernatant liquid was filtered through a 0.45 μm polyethersulfone (PES) membrane. The filtrates were tested for pH value, P, and Pb content. The centrifugal precipitates were collected and dried at 55°C for 24 h to determine the biomass, XRD, and SEM characterization.

A parallel experiment was also performed to detect the Pb removal capacity by different phosphates. The initial Pb concentration was also adjusted to 1,000 mg/L. Five treatments were performed in this experiment without Rho, that is, Pb, Fe-P + Pb, Al-P + Pb, Ca-P + Pb, and PG + Pb. After shaking for 6 days at 180 rpm, 28°C, the liquid was filtered through a 0.45-μm polyethersulfone (PES) membrane and prepared for the test of P and Pb content. All treatments were performed with three replicates.

### Extraction of EPS

After incubation, the supernatant was collected and centrifuged twice at 12,000 rpm at 4°C for 20 min. Then, a 3-fold volume of anhydrous ethanol was mixed with the supernatant to rest for 48 h at 4°C to collect extracellular secretions. Last, the collected crude extracts were transferred into 3,500 molecular dialysis bags and dipped in pure water for 72 h. The pure water was changed twice every 24 h. The last extracts were transferred into a 2-ml centrifuge tube and freeze-dried (Jiang et al., 2020).

### Instrumentation

The pH value in each treatment was determined by a pH meter (FE20, Mettler-Toledo, Int. Inc.). The soluble P and Pb concentrations were analyzed *via* ICP-OES (PerkinElmer Avio 200). A calibration curve of P and Pb (1, 5, 10, 20, 50, and 100 mg/L) was performed by the phosphorus and lead standard ((National Center of Analysis and Testing for Nonferrous Metals and Electronic Materials, China), respectively. The R square value of the external standard curves was 0.999.

The mineralogical characterization of the precipitates was examined using a Rigaku D/Max-2500 X-ray diffractometer (Cu-Kα; 36 kV; 20 mA; scanned from 5° to 60° at a speed of 4° s^−1^). The XRD patterns were analyzed by MDI Jade 6.5 software for phase identification.

The morphology of Rho and minerals was observed by SEM (S4800 Hitachi), with an acceleration voltage of 3 kV. To enhance image quality, the samples were coated with a layer of gold for 1 min in a Hitachi E-1010 Sputter.

## Results

### Medium pH and dry Biomass in Rho

**FIGURE 1:**
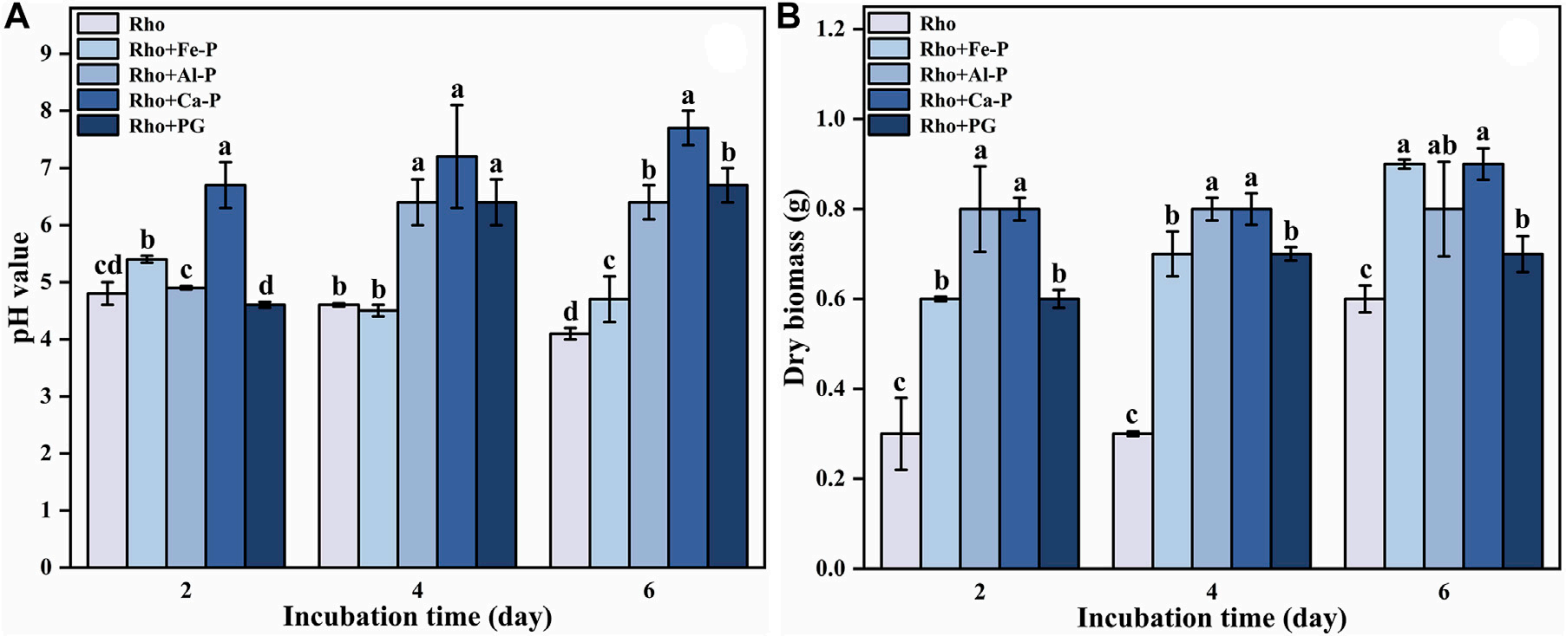
pH value **(A)** and dry biomass **(B)** in each treatment during the incubation time (2, 4, and 6 days). The error bars represent the standard deviations of three replicates. The significant differences among the treatments were identified by Tukey’s honestly significant difference test (*p* < 0.05) *via* one-way ANOVA.

The initial pH value in the medium was 5.7. After 2 days of incubation, the pH values of Rho and Rho + Fe-P treatments decreased to 4.8 and 5.4, respectively (Figure 1A). In Rho treatment, the pH value constantly decreased to 4.6 and 4.1 on days 4 and 6, respectively (Figure 1A). In Rho + Fe-P treatment, the pH value decreased to 4.5 on day 4 and increased to 4.7 on day 6 (Figure 1A). However, all of the pH values in Rho + Al-P, Rho + Ca-P, and Rho + PG treatments showed an increasing trend during the incubation time (Figure 1A). The pH value in Rho + Al-P treatment was 4.9, 6.4, and 6.4 on days 2, 4, and 6, respectively (Figure 1A). In Rho + Ca-P and Rho + PG treatments, consistent increases occurred in pH values from 6.7 to 7.7 and 4.6 to 6.7 during the incubation time, respectively (Figure 1A).

The dry biomass in Rho treatment was 0.3, 0.3, and 0.6 g on days 2, 4, and 6 (Figure 1B). In Rho + Fe-P treatment, the dry biomass increased from 0.6 g on day 2 to 0.7 and 0.9 g on days 4 and 6, respectively (Figure 1B). There was no discernible change in the dry biomass of Rho + Al-P during the incubation time, that is, 0.8 g (Figure 1B). In Rho + Ca-P and Rho + PG treatments, the dry biomass increased from 0.8 and 0.6 g to 0.9 and 0.7 g after 6 days of incubation, respectively (Figure 1B).

### P and Pb Concentration in the Medium

**FIGURE 2:**
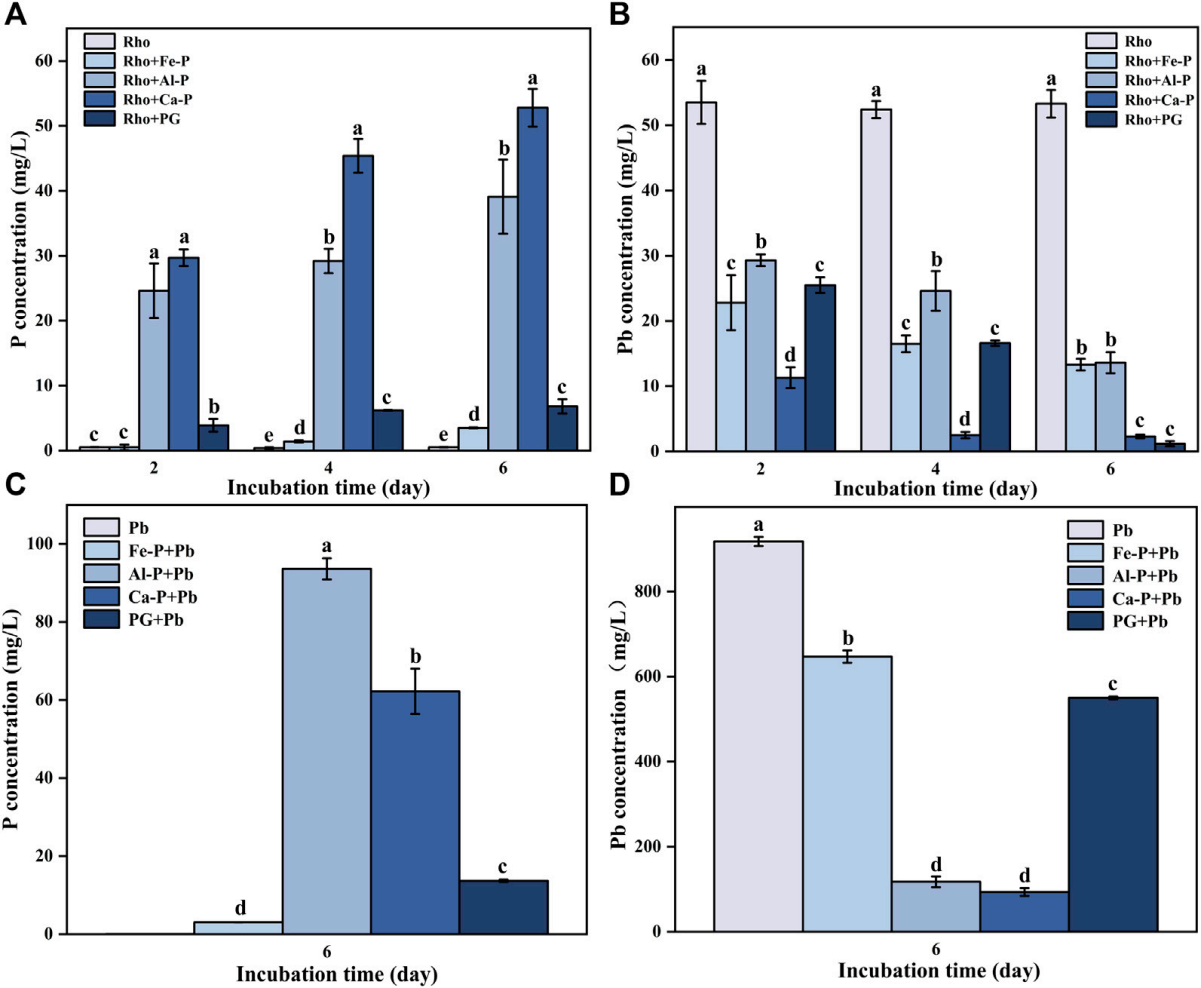
P concentration **(A,C)** and Pb concentration **(B,D)** in each treatment during the incubation time (2, 4, and 6 days). The error bars represent the standard deviations of three replicates. The significant differences among the treatments were identified by Tukey’s honestly significant difference test (*p* < 0.05) *via* one-way ANOVA.

The P contents in Rho and Rho + Fe-P treatments were lower, ranging from 0.4 to 0.5 mg/L and 0.5–3.5 mg/L, respectively, during the entire incubation time (Figure 2A). In Rho + Al-P and Rho + PG treatments, the P content was 24.6 and 3.9 mg/L on day 2, respectively (Figure 2A). After incubating for 4 and 6 days, the P concentration increased to 29.2 and 39.1 mg/L, and 6.2 and 6.8 mg/L in these two treatments, respectively (Figure 2A). Rho + Ca-P had the highest P content compared with other treatments, that is, 29.7, 45.4, and 52.8 mg/L on days 2, 4, and 6, respectively (Figure 2A).

The initial Pb concentration in the PDB medium was 1,000 mg/L. The Pb contents in Rho treatment were 53.5, 52.4, and 53.3 mg/L after 2, 4, and 6 days of incubation, respectively (Figure 2B). In Rho + Fe-P and Rho + Al-P treatments, the Pb content decreased from 22.8 to 29.3 and 13.3 to 13.6 mg/L after 6 days of incubation, respectively (Figure 2B). In Rho + Ca-P treatment, the Pb concentration decreased from 11.3 to 2.5 and 2.3 mg/L after 4 and 6 days of incubation, respectively (Figure 2B). Rho + PG had the lowest Pb content in each treatment, that is, 1.2 mg/L after 6 days of incubation (Figure 2B).

Results of our parallel experiment indicated that the P content in Pb, Fe-P + Pb, Al-P + Pb, Ca-P + Pb, and PG + Pb treatments reached 0.02, 3.01, 93.6, 62.2, and 13.7 mg/L after 6 days of incubation (Figure 2C). In Pb treatment, the Pb concentration had the highest value of 917.6 mg/L compared with other treatments (Figure 2D). Meanwhile, the Pb concentration in Fe-P + Pb, Al-P + Pb, Ca-P + Pb, and PG + Pb treatments significantly decreased to 647.1, 117.4, 93.6, and 550.1 mg/L after 6 days of incubation, respectively (Figure 2D).

### Pb Removal Ratio and EPS Secreted by Rho

**FIGURE 3:**
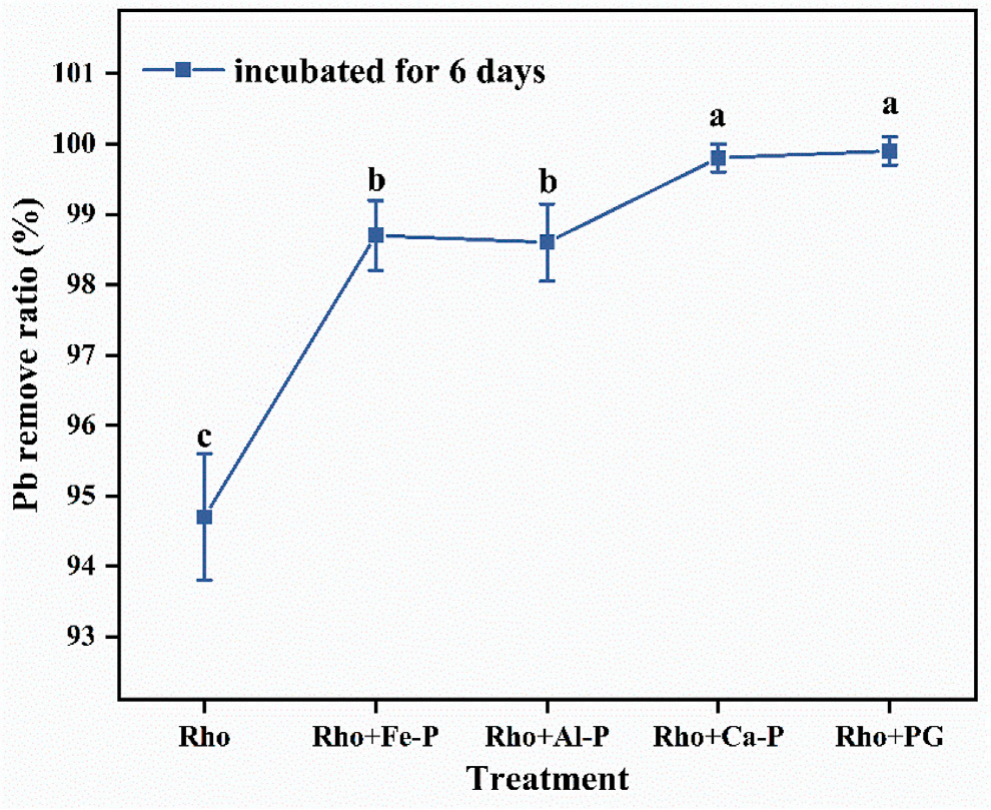
Removal ratio of Pb in each treatment after 6 days of incubation. The error bars represent the standard deviations of three replicates. The significant differences among the treatments were identified by Tukey’s honestly significant difference test (*p* < 0.05) *via* one-way ANOVA.

**FIGURE 4:**
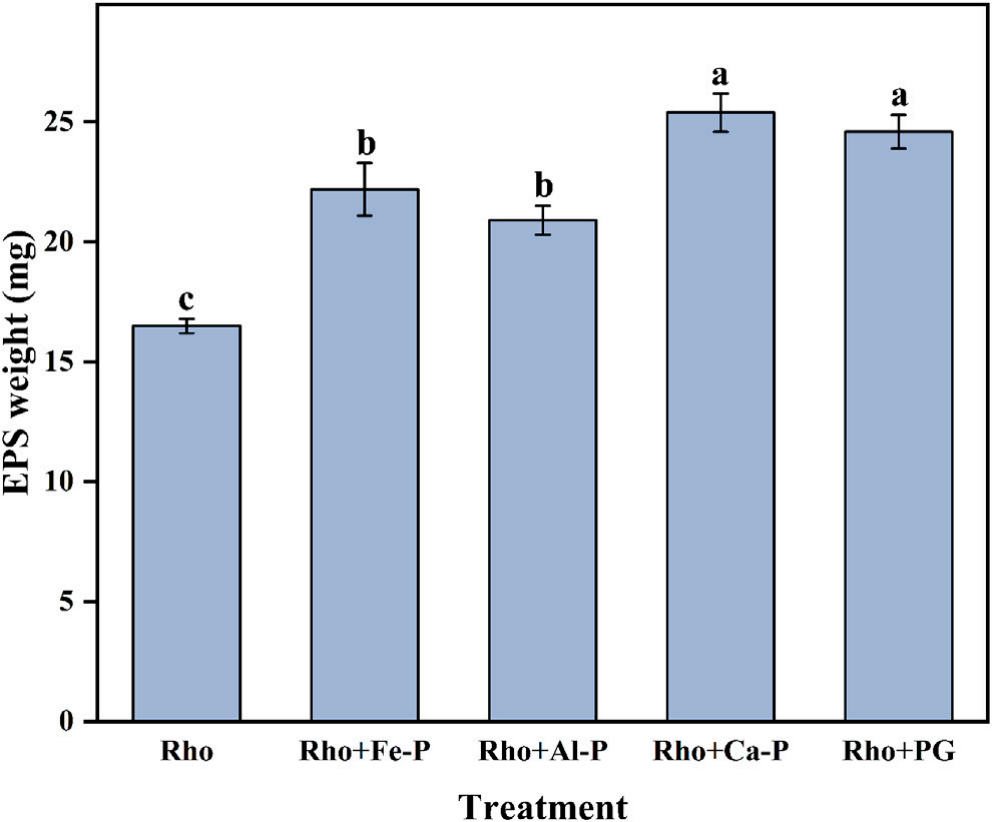
EPS weight in each treatment after 6 days of incubation. The error bars represent the standard deviations of three replicates. The significant differences among the treatments were identified by Tukey’s honestly significant difference test (*p* < 0.05) *via* one-way ANOVA.

The Pb removal ratios in Rho and Pb treatment were 94.7 and 8.2% after 6 days of incubation, respectively (Figure 3A,B). Compared with Rho, Rho + Fe-P, Rho + Al-P, Rho + Ca-P, and Rho + PG treatments had significantly higher Pb removal ratios of 98.7, 98.6, 99.8, and 99.9%, respectively, on day 6 (Figure 3A). Although phosphate species increased the Pb removal ratio, it was still lower than Rho treatment, that is, 35.3, 88.3, 90.6, and 44.9%, in Fe-P + Pb, Al-P + Pb, Ca-P + Pb, and PG + Pb treatments, respectively (Figure 3B). The EPS weight in Rho treatment was 16.5 mg after 6 days of incubation (Figure 4). In Rho + Fe-P, Rho + Al-P, Rho + Ca-P, and Rho + PG treatments, the EPS content significantly increased to 22.2, 20.9, 25.4, and 24.6 mg, respectively (Figure 4).

### XRD and SEM-EDS Analysis

**FIGURE 5:**
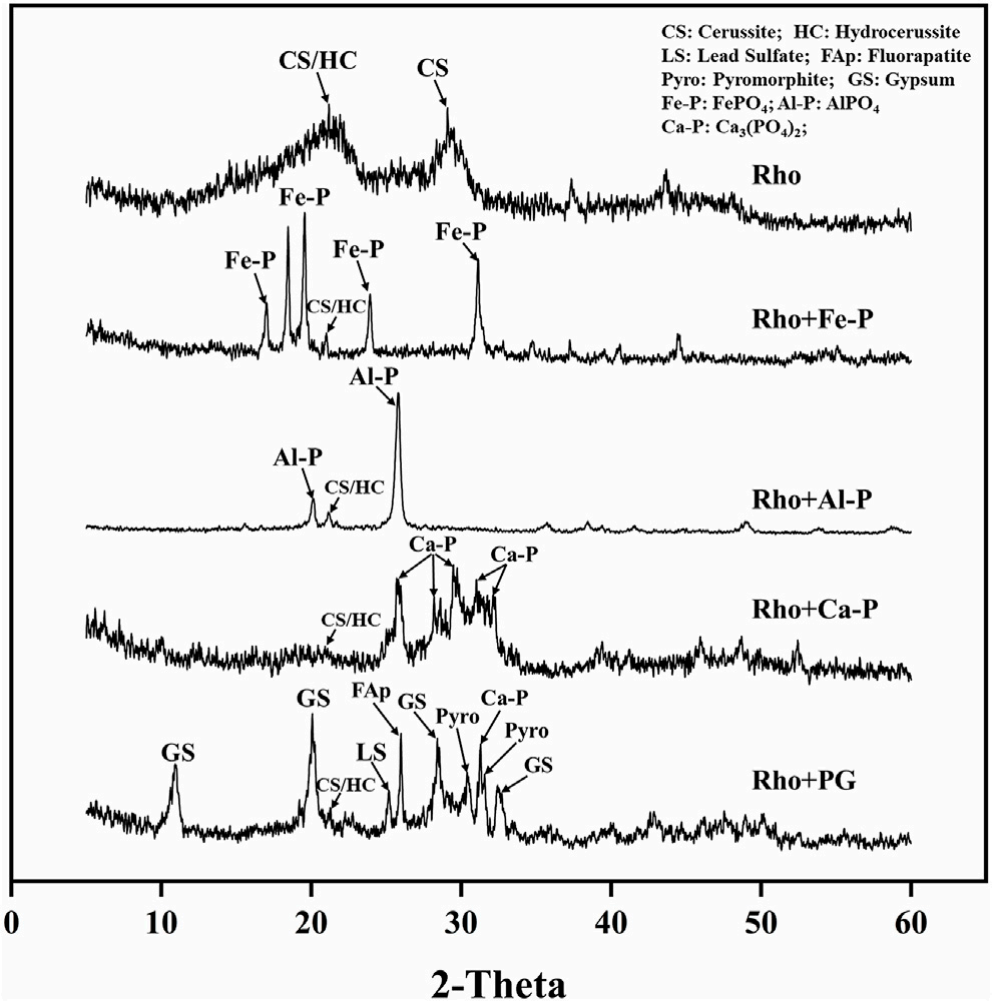
XRD patterns of precipitation in each treatment after 6 days of incubation.

The XRD patterns showed the mineralogical characteristics of precipitates in each treatment after 6 days of incubation (Figure 5). The peak of cerussite/hydrocerussite (21^o^) can be observed in each treatment (Figure 5). In addition, the strong peak of cerussite (29.2^o^) was also clearly observed in the Rho treatment (Figure 5). The strong peaks of Fe-P (17.0, 22.3, 23.9, 31.1°) and Al-P (20 and 25^o^) appeared in Rho + Fe-P and Rho + Al-P treatments, respectively (Figure 5). In Rho + Ca-P treatment, the peaks at 25.8, 28.3, 29.5, 31.0, and 32.2^o^ stand for the mineral of Ca-P (Figure 5). However, the formed mineral was more complex in Rho + PG treatment. Except for cerussite/hydrocerussite, both the peaks of fluorapatite (25.9^o^) and Ca-P (32.2^o^) were observed in Rho + PG treatment (Figure 5). In addition, the dominant gypsum peaks (11.5, 20.8, 29.0, and 33.3^o^) were also identified in Rho + PG treatment (Figure 5). More importantly, only the newly formed Pb minerals of pyromorphite (30.7, 31.7^o^) and lead sulfate (24.7^o^) were detected in this treatment (Figure 5).

**FIGURE 6:**
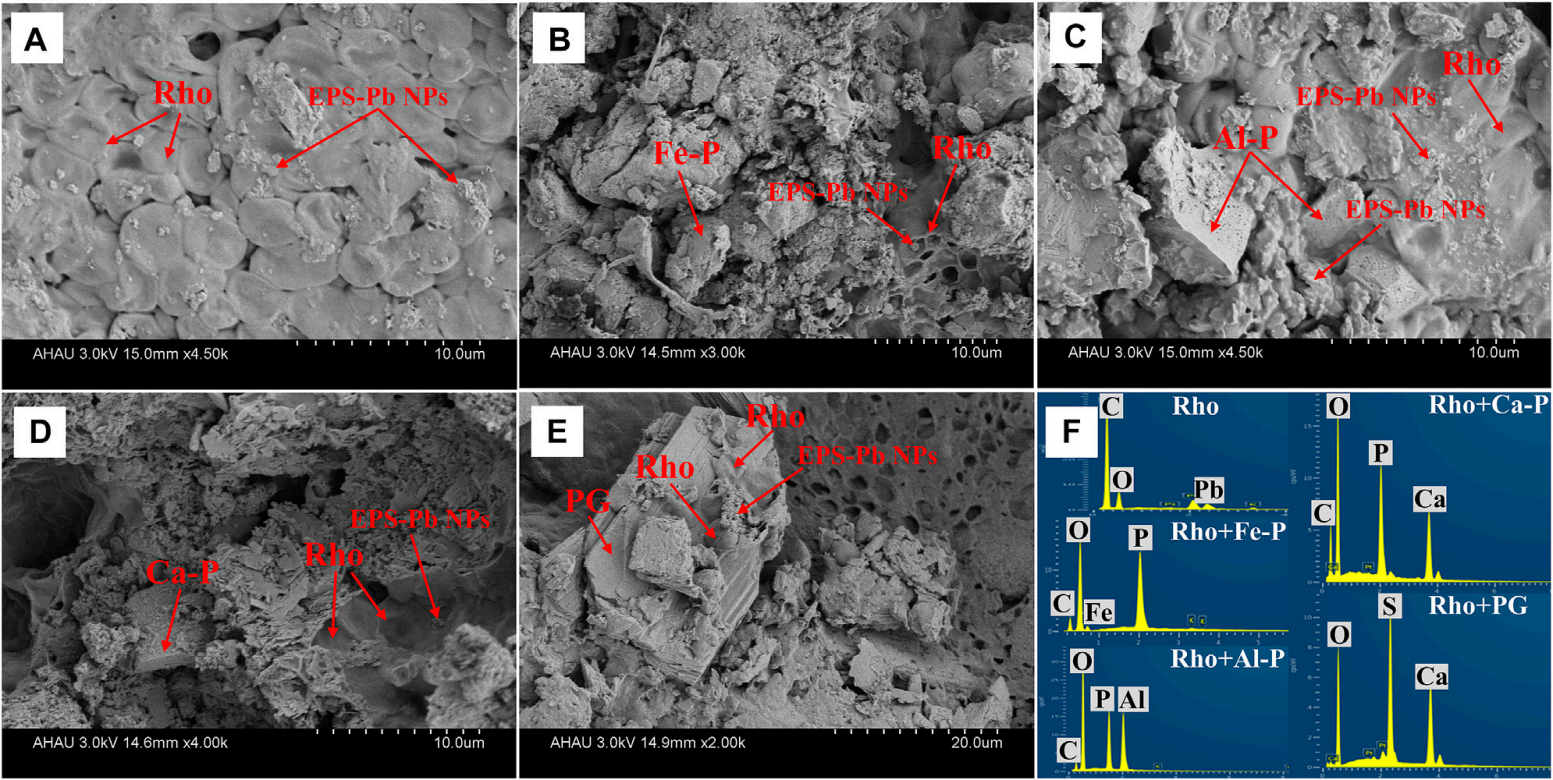
SEM image data in Rho **(A)**, Rho + Fe-P **(B)**, Rho + Al-P **(C)**, Rho + Ca-P **(D)**, and Rho + PG **(E)** treatments after 6 days of incubation. The EDS results **(F)** in each treatment after 6 days of incubation. The Rho and EPS-Pb can be observed in each treatment. Fe-P: ferric phosphate (FePO_4_); Al-P: aluminum phosphate (Al-P, AlPO_4_); Ca-P: tricalcium phosphate (Ca_3_(PO_4_)_2_).

The SEM-EDS images of the morphologies of Rho and different phosphate species are shown in Figure 6. As can be seen, EPS-Pb was formed in all treatments through the combination of extracellular polymeric substances (EPS) and Pb cations (Figure 6).

## Discussion

The fungus has a huge potential in the remediation of Pb contaminations in water, especially *via* the combination of phosphate (Ayangbenro & Babalola, 2017; Liang & Gadd, 2017). Our findings highlight the great potential of Rho for the remediation of Pb-contaminated water by the combination of phosphate. Co-application of Rho and various phosphates (Fe, Al, Ca-P, and PG) can remove >98% Pb cations, and the highest rate (99.9%) of Pb removal occurred in Rho + PG treatment (Figure 3). These results emphasize the superiority of Rho compared with other fungi (e.g., PSF) in terms of Pb remediation. In the case of PSF–*Penicillium oxalicum* (*P. oxalicum*), the Pb remediation by *P. oxalicum* was significantly limited by the high Pb toxicity (Tian et al., 2018b). Although *P. oxalicum* can resist ∼1,000 mg/L Pb level, the organic acid secretion ability was significantly limited, especially for oxalic acid (Tian et al., 2019). Under 1,500 mg/L Pb stresses, *P. oxalicum* had the lowest fungal biomass and almost lost its secretion ability of oxalic acid (Tian et al., 2019). In contrast, Rho has a higher Pb tolerance than *P. oxalicum*. Rho can still be highly active under 2000 mg/L Pb stress due to the high secretion of EPS and functional structure of vesicles (Jiang et al., 2020). More importantly, the higher Pb level even stimulates the Rho activity (Jiang et al., 2020). Hence, Rho would be more effective in Pb remediation with phosphate.

Previous works confirmed the efficacy of combined application of phosphate and fungi to enhance Pb remediation (Ma et al., 1995; Tian et al., 2018a; Traina & Laperche, 1999). Herein, phosphate-released P can react with Pb to form insoluble pyromorphite (Li et al., 2016). An acidic environment (lower than 4.5) can significantly promote the release of P from phosphate, especially for Ca-P (Tian et al., 2021b). In this study, the pH value in Rho was consistently higher than 4.5 during the incubation time, that is, pH 5–7 (Figure 1A). Hence, the highest P content released by Rho reached ∼55 mg/L but was much lower than PSF (∼307 mg/L) (Li et al., 2016). Although PSF has a higher P release ratio than Rho, the Pb removal ratio between these two fungi is similar (98%) (Figure 3). Hence, the Pb remediation in Rho + phosphate treatment is not limited by the lower content of P, but *via* the EPS pathway. The addition of phosphate can support the production of EPS by Rho in Pb remediation, rather than the released P.

The mechanisms between Rho and PSF are different due to the secretion of secondary metabolites. The organic acids, such as oxalic acid secreted by PSF, not only promote the release of P but also directly bind Pb to form insoluble lead oxalate (Strasser et al., 1994; Gadd et al., 2014; Tian et al., 2021a). The secretion of oxalic acid usually dominates the Pb immobilization process *via* the formation of new Pb minerals, such as pyro and lead oxalate (Li et al., 2016; Tian et al., 2018b). However, no new Pb minerals appeared in Rho + phosphate treatment except for PG. The secretion of large amounts of EPS is the main mechanism of Pb immobilization of Rho and phosphate (Fe, Al, Ca-P) treatments. Our SEM-EDS results also confirmed the large amounts of EPS-Pb formed in each treatment (Figure 5).

EPS has strong adsorption and binding capacity for Pb cations due to the complex molecular composition (Duda-Chodak et al., 2012). The high Pb level can stimulate EPS secretion by Rho to increase toxicity tolerance (Jiang et al., 2020). The formed EPS-Pb can reduce the Pb toxicity and promote the normal proliferation of Rho. However, the secretion of EPS by Rho was influenced by different factors (Gadd, 1990; Naveed et al., 2019). Our results indicated that Ca-P and PG had significantly higher contents of EPS than Fe-P and Al-P (Figure 4). In addition, the dissolution of phosphate can also release Ca, Fe, and Al cations (Li et al., 2017). These cations could also affect the Pb remediation *via* the EPS production, cell aggregation, and cell surface charges (Sheng et al., 2010). Although the addition of phosphate can promote the secretion of EPS by Rho in Pb remediation, only Rho + Ca-P and Rho + PG have the lowest Pb content (Figure 2B). Compared with divalent cations (e.g., Ca^2+^), the trivalent cations (e.g., Fe^3+^ and Al^3+^) are more competitive for heavy metal cations in binding with EPS (Yan et al., 2017). In addition, the Ca^2+^ on the EPS surface is more easily displaced by other greater affinity cations (e.g., Cd^2+^, Pb^2+^, and Cu^2+^) (Naveed et al., 2019). Therefore, the calcium-bound phosphate (Ca-P and PG) is more suitable for the remediation of Pb by Rho.

In this study, PG had the highest efficacy for Pb remediation by Rho. Except for EPS-Pb, the formed pyro only occurred in Rho + PG treatment (Figure 5). Since PG is a by-product of the dissolution of geological apatite by strong chemical acid, it contains large amounts of water-soluble P (Vinnichenko & Riazanov, 2020). Soluble P can react with lead cations before the combination of EPS and Pb, hence contributing to the formation of pyro. In addition, PG also contained large amounts of sulfate in the form of gypsum (Saadaoui et al., 2017). This sulfate can also react with Pb to form insoluble lead sulfate, which contributes to the remediation of Pb in water (Li et al., 2018). Hence, PG is more effective than Fe-P, Al-P, and Ca-P in Pb remediation by Rho. Both the formation of lead minerals and EPS-Pb by PG and Rho contribute to Pb immobilization. Therefore, a feasible approach for PG recyclability can happen through its combination with Rho for Pb remediation.

## Conclusion

This study concludes on the co-application of Rho and phosphate to significantly promote the Pb remediation in water. The secretion of EPS by Rho dominated the Pb removal *via* the formation of EPS-Pb, particularly in combination with Ca-P and PG. In addition, PG revealed the highest removal rate of Pb through the formation of pyro and lead sulfate. Altogether, our findings suggest that the combined application of PG and Rho is an economical and efficient approach for Pb remediation.

## Data Availability

The original contributions presented in the study are included in the article/Supplementary Material, further inquiries can be directed to the corresponding authors.

## References

[B1] Ayangbenro A. Babalola O. (2017). A New Strategy for Heavy Metal Polluted Environments: A Review of Microbial Biosorbents. Ijerph 14, 94. 10.3390/ijerph14010094 PMC5295344 28106848

[B2] Cao X. Wahbi A. Ma L. Li B. Yang Y. (2009). Immobilization of Zn, Cu, and Pb in Contaminated Soils Using Phosphate Rock and Phosphoric Acid. J. Hazard. Mater. 164, 555–564. 10.1016/j.jhazmat.2008.08.034 18848390

[B3] Coutinho F. P. Felix W. P. Yano-Melo A. M. (2012). Solubilization of Phosphates *In Vitro* by *Aspergillus* Spp. And *Penicillium* Spp. Ecol. Eng. 42, 85–89. 10.1016/j.ecoleng.2012.02.002

[B4] Duda-Chodak A. Tarko T. Milotta K. (2012). Applicability of Different Kinds of Yeast Biomass to lead Removal from Water. J. Elemntol. 17, 7–18. 10.5601/jelem.2012.17.1.01

[B5] Frisvad J. C. Møller L. L. H. Larsen T. O. Kumar R. Arnau J. (2018). Safety of the Fungal Workhorses of Industrial Biotechnology: Update on the Mycotoxin and Secondary Metabolite Potential of Aspergillus niger, Aspergillus oryzae, and Trichoderma Reesei. Appl. Microbiol. Biotechnol. 102, 9481–9515. 10.1007/s00253-018-9354-1 30293194 PMC6208954

[B6] Gadd G. M. Bahri-Esfahani J. Li Q. Rhee Y. J. Wei Z. Fomina M. (2014). Oxalate Production by Fungi: Significance in Geomycology, Biodeterioration and Bioremediation. Fungal Biol. Rev. 28, 36–55. 10.1016/j.fbr.2014.05.001

[B7] Gadd G. M. (1990). “Fungi and Yeasts for Metal Accumulation,” in Microbial Mineral Recovery. Editors Ehrlich H. L. Brierley C. L. (New York: McGraw-Hill Publishing), 249–276.

[B8] Gadd G. M. (1993). Interactions of Fungi with Toxic Metals. New Phytol. 124, 25–60. 10.1111/j.1469-8137.1993.tb03796.x

[B9] Gientka I. Blazejak S. Błażejak S. Stasiak-Różańska L. Chlebowska-Śmigiel A. (2015). Exopolysaccharides from Yeast: Insight into Optimal Conditions for Biosynthesis, Chemical Composition and Functional Properties – Review. Acta Sci. Pol. Technol. Aliment. 14, 283–292. 10.17306/j.afs.2015.4.29 28068035

[B10] Hou D. Ding Z. Li G. Wu L. Hu P. Guo G. (2018). A Sustainability Assessment Framework for Agricultural Land Remediation in China. Land. Degrad. Dev. 29, 1005–1018. 10.1002/ldr.2748

[B11] Hou D. Qi S. Zhao B. Rigby M. O'Connor D. (2017). Incorporating Life Cycle Assessment with Health Risk Assessment to Select the 'greenest' Cleanup Level for Pb Contaminated Soil. J. Clean. Prod. 162, 1157–1168. 10.1016/j.jclepro.2017.06.135

[B12] Jiang Z. Wang T. Sun Y. Nong Y. Tang L. Gu T. (2020). Application of Pb(II) to Probe the Physiological Responses of Fungal Intracellular Vesicles. Ecotoxicology Environ. Saf. 194, 110441. 10.1016/j.ecoenv.2020.110441 32155484

[B13] Kaludjerovic-Radoicic T. Raicevic S. (2008). *In Situ* Lead Stabilization Using Natural and Synthetic Apatite. CI&CEQ 14, 269–271. 10.2298/ciceq0804269k

[B14] Kot A. M. Błażejak S. Kieliszek M. Gientka I. Bryś J. Reczek L. (2019b). Effect of Exogenous Stress Factors on the Biosynthesis of Carotenoids and Lipids by Rhodotorula Yeast Strains in media Containing Agro-Industrial Waste. World J. Microbiol. Biotechnol. 35, 157. 10.1007/s11274-019-2732-8 31576445 PMC6773817

[B15] Kot A. M. Błażejak S. Kieliszek M. Gientka I. Bryś J. (2019a). Simultaneous Production of Lipids and Carotenoids by the Red Yeast Rhodotorula from Waste Glycerol Fraction and Potato Wastewater. Appl. Biochem. Biotechnol. 189, 589–607. 10.1007/s12010-019-03023-z 31073981 PMC6754821

[B16] Li J. Jiang Z. Chen S. Wang T. Jiang L. Wang M. (2019). Biochemical Changes of Polysaccharides and Proteins within EPS under Pb(II) Stress in *Rhodotorula Mucilaginosa* . Ecotoxicology Environ. Saf. 174, 484–490. 10.1016/j.ecoenv.2019.03.004 30856560

[B17] Li Z. Su M. Duan X. Tian D. Yang M. Guo J. (2018). Induced Biotransformation of lead (II) by Enterobacter Sp. In SO4-PO4-Cl Solution. J. Hazard. Mater. 357, 491–497. 10.1016/j.jhazmat.2018.06.032 29940467

[B18] Li Z. Su M. Tian D. Tang L. Zhang L. Zheng Y. (2017). Effects of Elevated Atmospheric CO2 on Dissolution of Geological Fluorapatite in Water and Soil. Sci. Total Environ. 599-600, 1382–1387. 10.1016/j.scitotenv.2017.05.100 28531916

[B19] Li Z. Wang F. Bai T. Tao J. Guo J. Yang M. (2016). Lead Immobilization by Geological Fluorapatite and Fungus *Aspergillus niger* . J. Hazard. Mater. 320, 386–392. 10.1016/j.jhazmat.2016.08.051 27585270

[B20] Liang X. Gadd G. M. (2017). Metal and Metalloid Biorecovery Using Fungi. Microb. Biotechnol. 10, 1199–1205. 10.1111/1751-7915.12767 28696059 PMC5609339

[B21] Ma Q. Y. Logan T. J. Traina S. J. (1995). Lead Immobilization from Aqueous Solutions and Contaminated Soils Using Phosphate Rocks. Environ. Sci. Technol. 29, 1118–1126. 10.1021/es00004a034 22176421

[B22] Naveed S. Li C. Lu X. Chen S. Yin B. Zhang C. (2019). Microalgal Extracellular Polymeric Substances and Their Interactions with Metal(loid)s: A Review. Crit. Rev. Environ. Sci. Technol. 49, 1769–1802. 10.1080/10643389.2019.1583052

[B23] Rahbar Saadat Y. Yari Khosroushahi A. Pourghassem Gargari B. (2021). Yeast Exopolysaccharides and Their Physiological Functions. Folia. Microbiol. 66, 171–182. 10.1007/s12223-021-00856-2 33604744

[B24] Rhee Y. J. Hillier S. Gadd G. M. (2012). Lead Transformation to Pyromorphite by Fungi. Curr. Biol. 22, 237–241. 10.1016/j.cub.2011.12.017 22245002

[B25] Saadaoui E. Ghazel N. Ben Romdhane C. Massoudi N. (2017). Phosphogypsum: Potential Uses and Problems - a Review. Int. J. Environ. Stud. 74, 558–567. 10.1080/00207233.2017.1330582

[B26] Shen Z. Tian D. Zhang X. Tang L. Su M. Zhang L. (2018). Mechanisms of Biochar Assisted Immobilization of Pb2+ by Bioapatite in Aqueous Solution. Chemosphere 190, 260–266. 10.1016/j.chemosphere.2017.09.140 28992478

[B27] Sheng G.-P. Yu H.-Q. Li X.-Y. (2010). Extracellular Polymeric Substances (EPS) of Microbial Aggregates in Biological Wastewater Treatment Systems: a Review. Biotechnol. Adv. 28, 882–894. 10.1016/j.biotechadv.2010.08.001 20705128

[B28] Strasser H. Burgstaller W. Schinner F. (1994). High-yield Production of Oxalic Acid for Metal Leaching Processes byAspergillus niger. FEMS. Microbiol. Lett. 119, 365–370. 10.1111/j.1574-6968.1994.tb06914.x 8050718

[B29] Tian D. Jiang Z. Jiang L. Su M. Feng Z. Zhang L. (2019). A New Insight into lead (II) Tolerance of Environmental Fungi Based on a Study of *Aspergillus niger* and *Penicillium oxalicum* . Environ. Microbiol. 21, 471–479. 10.1111/1462-2920.14478 30421848

[B30] Tian D. Lai Z. Zou X. Guo C. Tang L. Su M. (2018a). A Contrast of lead Immobilization via Bioapatite under Elevated CO2 between Acidic and Alkaline Soils. Soil Use. Manage. 34, 542–544. 10.1111/sum.12448

[B31] Tian D. Su M. Zou X. Zhang L. Tang L. Geng Y. (2021a). Influences of Phosphate Addition on Fungal Weathering of Carbonate in the Red Soil from Karst Region. Sci. Total Environ. 755, 142570. 10.1016/j.scitotenv.2020.142570 33035850

[B32] Tian D. Wang L. Hu J. Zhang L. Zhou N. Xia J. (2021b). A Study of P Release from Fe-P and Ca-P via the Organic Acids Secreted by *Aspergillus niger* . J. Microbiol. 59, 819–826. 10.1007/s12275-021-1178-5 34382148

[B33] Tian D. Wang W. Su M. Zheng J. Wu Y. Wang S. (2018b). Remediation of lead-contaminated Water by Geological Fluorapatite and Fungus Penicillium oxalicum. Environ. Sci. Pollut. Res. 25, 21118–21126. 10.1007/s11356-018-2243-4 29770937

[B34] Traina S. J. Laperche V. (1999). Contaminant Bioavailability in Soils, Sediments, and Aquatic Environments. Proc. Natl. Acad. Sci. 96, 3365–3371. 10.1073/pnas.96.7.3365 10097045 PMC34276

[B35] Vinnichenko V. Riazanov A. (2020). Environmental Problems of Phosphogypsum Utilization. Kem 864, 108–114. 10.4028/www.scientific.net/kem.864.108

[B36] Yan P. Xia J.-S. Chen Y.-P. Liu Z.-P. Guo J.-S. Shen Y. (2017). Thermodynamics of Binding Interactions between Extracellular Polymeric Substances and Heavy Metals by Isothermal Titration Microcalorimetry. Bioresour. Technol. 232, 354–363. 10.1016/j.biortech.2017.02.067 28249189

[B37] Zeng G. Wan J. Huang D. Hu L. Huang C. Cheng M. (2017). Precipitation, Adsorption and Rhizosphere Effect: The Mechanisms for Phosphate-Induced Pb Immobilization in Soils-A Review. J. Hazard. Mater. 339, 354–367. 10.1016/j.jhazmat.2017.05.038 28668753

